# The combination of nonthyroidal illness syndrome and renal dysfunction further increases mortality risk in patients with acute myocardial infarction: a prospective cohort study

**DOI:** 10.1186/s12872-019-1027-1

**Published:** 2019-03-04

**Authors:** Jun-Wei Wang, Ying Ren, Zhi-Gang Lu, Jing Gao, Cui-Chun Zhao, Lian-Xi Li, Meng Wei

**Affiliations:** 1 Department of VIP, Shanghai Jiao Tong University Affiliated Sixth People’s Hospital, 600 Yishan Road, Shanghai, 200233 China; 2 Department of Cardiology, Shanghai Jiao Tong University Affiliated Sixth People’s Hospital, 600 Yishan Road, Shanghai, 200233 China; 3 Department of Endocrinology and Metabolism, Shanghai Jiao Tong University Affiliated Sixth People’s Hospital; Shanghai Diabetes Institute; Shanghai Key Laboratory of Diabetes Mellitus; Shanghai Clinical Center for Diabetes; Shanghai Key Clinical Center for Metabolic Disease, 600 Yishan Road, Shanghai, 200233 China

**Keywords:** Nonthyroidal illness syndrome, Renal insufficiency, Acute myocardial infarction, Cardio-renal-nonthyroidal illness syndrome

## Abstract

**Background:**

Both nonthyroidal illness syndrome and renal dysfunction are associated with increased mortality risk in acute myocardial infarction (AMI). However, it is unclear whether combined NTIS and renal dysfunction further increase mortality risk. Therefore, our aim is to investigate whether combined NTIS and renal dysfunction further increases mortality risk in patients with acute myocardial infarction (AMI).

**Methods:**

A total of 1295 inpatients with AMI were divided into normal group (*n* = 692), NTIS group (*n* = 139), renal dysfunction group (*n* = 304), and combined NTIS and renal dysfunction group (*n* = 160). Heart function, in-hospital, all-cause and cardiovascular mortality were compared among the four groups.

**Results:**

After adjustment for age and sex, left ventricular ejection fraction was significantly lower in the combined group (48 ± 11%) than in the NTIS group (52 ± 10%, *P* = 0.017), the renal dysfunction group (52 ± 10%, *P* = 0.001) and the normal group (56 ± 8%, *P* < 0.001). After controlling for confounding factors, compared with the normal group, the NTIS and the renal dysfunction group represented higher risks of in-hospital mortality (OR: 3.643, *P* = 0.028; OR:3.135, *P* = 0.042, respectively), all-cause mortality (HR: 2.138, *P* = 0.007; HR: 2.050, *P* = 0.003, respectively), and cardiovascular mortality (HR:2.134, *P* = 0.042; HR:2.237, *P* = 0.010, respectively). Compared to those in the NTIS and the renal dysfunction group, the patients in the combined group showed a further increased risk for in-hospital mortality (OR:2.916, *P* = 0.039; OR:2.487, *P* = 0.036, respectively), all-cause mortality (HR: 1.939, *P* = 0.015; HR: 2.020, *P* = 0.002, respectively) and cardiovascular mortality (HR:2.420, *P* = 0.010; HR:2.303, *P* = 0.002, respectively).

**Conclusions:**

Both NTIS and renal dysfunction increase short-term in-hospital mortality, and long-term all-cause and cardiovascular mortality risk in patients with AMI. Furthermore, the coexistence of NTIS and renal dysfunction presents further increased mortality risk in AMI patients.

**Electronic supplementary material:**

The online version of this article (10.1186/s12872-019-1027-1) contains supplementary material, which is available to authorized users.

## Background

Ischemic heart disease will be the foremost cause of morbidity and mortality worldwide [1, 2]. As the first manifestation of ischemic heart disease, acute myocardial infarction (AMI) can cause acute alteration in thyroid function, referred to as “nonthyroidal illness syndrome (NTIS)”, which is characterized by decreased free T3 levels (FT3) with normal or low free thyroxine (FT4) and thyroid-stimulating hormone (TSH) levels [3, 4]. Currently, a growing number of studies have demonstrated that NTIS is an independent predictor of mortality in patients with heart failure and IHD [5–8]. For example, Iervasi et al. [7] reported that patients with NTIS had 1.6 times the risk of cardiovascular mortality than euthyroid patients. Iglesias et al. [9] also found that FT_3_ was an independent predictor of cardiovascular mortality in patients aged over 65 years.

On the other hand, renal insufficiency has been related to unfavorable performance in patients with AMI, such as increased cardiovascular mortality risk, painless myocardial infarction and various electrolyte disorders [10–12]. For example, the Survival and Ventricular Enlargement (SAVE) Study [13] showed that eGFR < 45 ml/min/1.73 m^2^ should be particularly considered as a major risk factor for cardiovascular mortality after AMI. Furthermore, Anavekar et al. [12] found that even mild reduced eGFR (below 81 ml/min/1.73 m^2^) was also closely associated with an increased risk for cardiovascular complications after AMI. Therefore, renal dysfunction is a powerful predictor of cardiovascular events and cardiovascular mortality [14, 15]. Moreover, renal insufficiency is also a pronounced marker of cardiac function and correlates directly with survival in patients with heart disease [16]. The increased cardiovascular mortality in patients with renal insufficiency can be caused by cardio-renal syndrome, which is regarded as disorders of the heart and kidney whereby dysfunction in one organ may induce dysfunction of the other in acute or chronic conditions [17].

Although both renal dysfunction and NTIS have been extensively investigated in AMI, few studies have thoroughly examined their reciprocal relationship in AMI patients. In recent years, the relationship between cardio-renal syndrome and thyroid function causes for concern [18]. Because cardio-renal syndrome is related to increased mortality risk and NTIS may cause the deterioration of both renal and cardiac function [16, 19, 20], we hypothesize that the combination of renal dysfunction and NTIS may cause a steep increase in mortality risk in AMI patients. Therefore, our primary aims are to assess the effect of renal dysfunction and NTIS on cardiac function and mortality risk, and to investigate whether the coexistence of NTIS and renal dysfunction further increases the future mortality risk in AMI.

## Methods

**Fig. 1:**
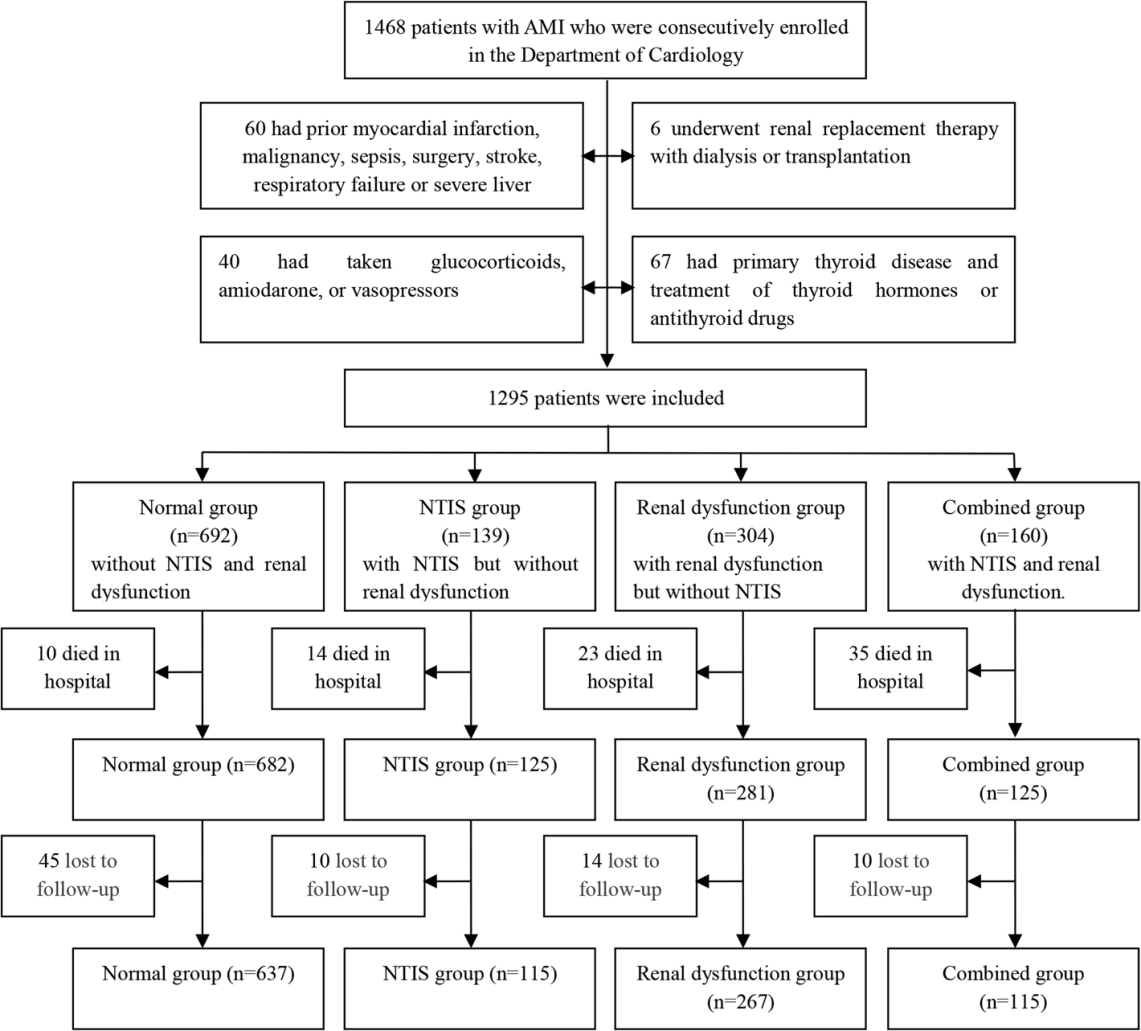
Enrollment and follow-up of subjects

From June 2005 to March 2013, 1468 patients with AMI aged 31 to 97 were consecutively enrolled in the Department of Cardiology of Shanghai Jiao Tong University Affiliated Sixth People’s Hospital. This observational prospective study got the approval of the ethics committee of Shanghai Jiao Tong University Affiliated Sixth People’s Hospital and written informed consent was obtained from each participant. The flow chart of enrollment of subjects in this study is presented in Fig. 1. Patients who presented with diseases or underwent medications that affected the measurement of thyroid profile or renal function were excluded from the study.

Ultimately, the remaining 1295 patients were included in the present study. NTIS was defined as FT3 level below the lower limit of the reference interval accompanied with FT4 and TSH in or below the reference range [3, 4, 6]. The reference intervals for thyroid function were as follows: FT3 3.1 to 6.8 pmol/l, FT4 12.0 to 22.0 pmol/l, and TSH 0.27 to 4.20 IU/ml. Renal function was evaluated by the estimated glomerular filtration rate (eGFR), which was calculated using the simplified MDRD formula: eGFR =175 × (Serum creatinine)^-1.154^ × (age)^-0.203^(× 0.742 if female) [21]. The cut-off value of renal dysfunction was 60 ml/min/1.73 m^2^.

Patients were divided into four groups based on the diagnosis of NTIS and renal dysfunction: normal group(*n* = 692), without NTIS and renal dysfunction; NTIS group(*n* = 139), with NTIS but without renal dysfunction; renal dysfunction group(*n* = 304), with renal dysfunction but without NTIS; combined group(*n* = 160), with NTIS and renal dysfunction.

The admitted 1295 subjects were interviewed to obtain their information on sex, age, histories of hypertension and diabetes mellitus, alcohol consumption, smoking habits, and medicine therapy. Physical examinations, including weight, height, and blood pressure, were performed for individuals according to our previous protocols [22–25]. Body mass index (BMI) was calculated as weight divided by height squared. Blood samples were taken for laboratory indicators within 24 h after admission. The laboratory measurements including serum creatinine (SCr), albumin (Alb), total triglycerides (TG), total cholesterol (TC), high-density lipoprotein cholesterol (HDL-C), low-density lipoprotein cholesterol (LDL-C), white blood cell count (WBC), hemoglobin (Hb), fasting plasma glucose (FPG), and C-reactive protein (CRP) were determined by standard laboratory protocols. NT-pro B-type natriuretic peptide (NT-pro BNP) and the thyroid function profile, including FT3, FT4, and TSH, were measured using a chemiluminescence technique (Cobas 6000; Roche Diagnostics GmbH, Mannheim, Germany). Coronary angiography was performed by standard Judkins techniques and patients with eGFR< 30 ml/min/1.73 m2 received iodixanol as contrast media and the others received iohexol. All patients received heparin during coronary angiography. Left ventricular ejection fraction (LVEF) was assessed by echocardiography (Acuson Sequoia 512 scanner; Siemens Medical Solutions, Mountain View, CA). Severe acute heart failure was regarded as Killip class>II [26]. Infarct type (NSTEMI vs STEMI), revascularization (percutaneous coronary intervention or coronary artery bypass graft), and in-hospital mortality was determined based on hospitalization records.

After discharge, the patients were followed up for 1 to 8.5 years (median 4 years). The data of overall and cardiovascular death were obtained annually from telephone interviews with patients or their relatives and hospital readmission records. Cardiovascular deaths required the documentation of myocardial infarction, cardiogenic shock, significant arrhythmia, severe heart failure, cerebrovascular events and pulmonary embolism. Sudden unexpected deaths which occurred outside the hospital were classified as cardiovascular deaths. Besides, overall and cardiovascular deaths did not include in-hospital deaths. The flow chart of follow-up of subjects is showed in Fig. 1. Finally, 79 patients (6.5%) were lost at follow-up.

The diagnosis of AMI was consistent with the guidelines of the ACC/AHA for the management of AMI in 1999 and 2007, including a dynamic rise and fall of Creatine-Kinase-MB isoform and Cardiac Troponin together with evidence of myocardial ischaemia [27, 28]. The criteria of hypertension, diabetes mellitus, smoking, and alcohol status were in accordance with description in our previous study [22–25].

The analyses of our data were conducted with SPSS 15.0 (SPSS Inc., Chicago, IL, USA). Normality was checked for continuous variables. Continuous variables were described as means with SDs or expressed as the median and inter-quartile range. One-way ANOVA with LSD or Kruskal-Wallis test were used to compare the differences of normally or non-normally distributed variables. Chi-square test was taken to compare the rates among four groups. Binary logistic regression was used to evaluate differences of categorical variables and to compare odds ratio (OR) of in-hospital mortality in the four groups. Linear regression was used to evaluate differences of continuous variables while controlling for other factors. Adjusted Kaplan-Meier survival curves were performed to estimate all-cause mortality and cardiovascular mortality. Cox proportional hazard regression analysis was applied to estimate hazard ratio (HR) of all-cause and cardiovascular death in the four groups. Three models were constructed: model 1 included adjustments for age, sex, smoking use, alcohol status, hypertension, diabetes mellitus, medical therapy and BMI; model 2 included additional adjustments for LVEF, Killip class, lg(NT-pro BNP) infarct type (NSTEMI vs STEMI) and revascularization; and model 3 had additional adjustment factors encompassing WBC, Hb, Alb, TG, TC, HDL-C,LDL-C, FPG and CRP. We also performed the sensitivity analysis by recalculating eGFR and regrouped which was based on CKD-EPI formula [29]. A *P*-value of < 0.05 was considered as statistically significant.

## Results

### Characteristics of study subjects

Enrollment and follow-up of subjects are completed in Fig. 1. The baseline characteristics of study subjects are presented in Table 1. The combined group was associated with higher prevalence of diabetes mellitus, higher WBC and CRP, less revascularization, and lower Hb compared to the NTIS, the renal dysfunction and the normal group after controlling for age and sex (all *P* < 0.05). In addition, older age, more female patients, higher CRP, higher prevalence of diabetes mellitus, and lower Hb and BMI were found in the NTIS group and the renal dysfunction group than in the normal group after adjustment for age and sex (all *P* < 0.05).

**Table 1:** Clinical characteristics of the study subjects

Variables	Normal (*n* = 692)	NTIS (*n* = 139)	Renal dysfunction (*n* = 304)	Combined (*n* = 160)	*P* value	*P* value*
Age (years)	65 ± 13	70 ± 12	75 ± 9	77 ± 10	< 0.001	< 0.001
Male(n,%)	536 (77.5%)	78 (56.1%)	147 (48.4%)	97 (60.6%)	< 0.001	0.001
Hypertension(n,%)	403 (58.2%)	83 (59.7%)	227 (74.7%)	107 (66.9%)	< 0.001	0.112
Diabetes mellitus (n,%)	181 (26.2%)	46 (33.1%)	111 (36.5%)	61 (38.1%)	0.001	0.001
Smoking(n,%)	452 (65.5%)	82 (59.0%)	150 (49.3%)	81 (50.6%)	< 0.001	0.083
Alcohol(n,%)	50 (7.3%)	5 (3.6%)	7 (2.3%)	6 (3.8%)	0.006	0.597
SBP (mmHg)	128 ± 22	123 ± 23	132 ± 24	127 ± 26	0.003	< 0.001
DBP (mmHg)	75 ± 13	70 ± 13	73 ± 13	72 ± 14	0.001	< 0.001
BMI (kg/m^2^)	24.24 ± 2.04	23.36 ± 2.53	23.55 ± 2.53	23.46 ± 2.66	< 0.001	0.03
WBC (×10^9^/l)	8.64 ± 3.69	9.71 ± 4.90	8.30 ± 3.98	9.98 ± 4.91	< 0.001	< 0.001
Hb (g/l)	139 ± 15	129 ± 16	124 ± 17	116 ± 20	< 0.001	< 0.001
Alb (g/l)	41 ± 6	38 ± 6	40 ± 5	37 ± 6	< 0.001	0.001
CRP (mg/l)	3.4 (1.6–6.5)	10.0 (4.8–21.3)	5.1 (3.5–8.0)	18.2 (8.8–33.0)	< 0.001	< 0.001
TG (mmol/l)	1.54 ± 0.91	1.37 ± 1.07	1.40 ± 0.80	1.30 ± 0.91	< 0.001	0.005
TC (mmol/l)	4.60 ± 1.15	4.64 ± 1.15	4.34 ± 1.14	4.24 ± 1.17	0.007	< 0.001
HDL-C(mmol/l)	1.05 (0.90–1.22)	1.05 (0.93–1.21)	1.06 (0.85–1.23)	1.03 (0.87–1.20)	0.361	0.245
LDL-C(mmol/l)	2.92 (2.34–3.55)	2.80 (2.23–3.55)	2.67 (2.09–3.44)	2.56 (2.02–3.25)	< 0.001	0.547
FPG (mmol/l)	6.98 ± 3.30	7.95 ± 3.65	7.10 ± 3.31	7.60 ± 3.07	0.003	0.007
FT3(pmol/l)	4.05 ± 0.55	2.60 ± 0.47	3.88 ± 0.53	2.52 ± 0.46	< 0.001	< 0.001
FT4(pmol/l)	16.15 ± 2.23	15.26 ± 2.06	16.52 ± 2.30	15.17 ± 2.53	< 0.001	< 0.001
TSH(mU/l)	1.30 (0.75–2.12)	1.26 (0.57–1.94)	1.47 (0.98–2.29)	1.25 (0.59–1.84)	< 0.001	0.004
sCr (μmol/l)	76 (65–88)	74 (60–89)	118 (102–142)	135 (109–178)	< 0.001	< 0.001
eGFR(ml/min/1.73m^2^)	89.69 ± 29.58	86.77 ± 28.15	44.03 ± 12.90	39.84 ± 14.50	< 0.001	< 0.001
Antiplatelet agents(n,%)	446 (64.6%)	93 (66.9%)	180 (59.2%)	96 (60.0%)	0.241	0.491
β-blockers(n,%)	379 (55.0%)	77 (55.4%)	217 (71.4%)	110 (68.8%)	< 0.001	0.005
LLDs (n,%)	462 (67.0%)	95 (68.3%)	158 (52.1%)	92 (57.5%)	< 0.001	0.040
ACEIs/ARBs(n,%)	363 (52.6%)	89 (64.0%)	140 (46.2%)	70 (43.8%)	0.001	0.133
CCBs (n,%)	81 (11.7%)	12 (8.6%)	39 (12.8%)	19 (11.9%)	0.649	0.922
Diuretics(n,%)	492 (71.2%)	116 (83.5%)	210 (69.1%)	114 (71.3%)	0.014	0.873
Revascularization during the present hospitalization(n,%)	539 (77.9%)	98 (70.5%)	163 (53.6%)	68 (42.5%)	< 0.001	< 0.001
Prior PCI or CABG (n,%)	24 (3.5%)	4 (2.9%)	6 (2.0%)	6 (3.8%)	0.604	0.622
Infarct type (NSTEMI n,%)	42 (6.1%)	11 (7.9%)	17 (5.6%)	12 (7.5%)	0.725	0.627

Values are expressed as mean ± SD,median with interquartile range or percentages

The *p* values were not adjusted for age and sex for the trend

The p values* were adjusted for age and sex for the trend

Revascularization included percutaneous coronary intervention (PCI) and coronary artery bypass graft (CABG)

### Comparison of cardiac function among the four groups

**Fig. 2:**
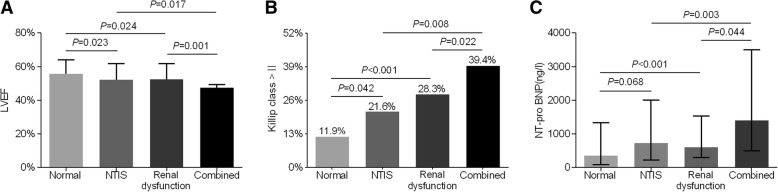
Comparison of cardiac function among the four groups at baseline. (**a**) Mean LVEF (%) among the four groups. (**b**) The percentage of Killip class>II (%) among the four groups. (**c**) Median NT-pro BNP (ng/l) among the four groups

As apparent from Fig. 2a, LVEF was significantly lower in the patients with combined NTIS and renal dysfunction (48 ± 11%) than in the patients with either NTIS (52 ± 10%, *P* = 0.017) or renal dysfunction (52 ± 10%, *P* = 0.001) and in the patients with normal FT3 and normal renal function (56 ± 8%, *P* < 0.001) after adjustment for age and sex (Fig. 2a). In addition, the percentage of Killip class>II in the combined group (39.4%) was markedly higher than that in the NTIS (21.6%, *P* = 0.008) and the renal dysfunction group (28.3%, *P* = 0.022) and that in the normal group (11.9%, *P* < 0.001) after controlling for age and gender variables (Fig. 2b). Furthermore, participants in the combined group had an obviously higher level of NT-pro BNP than participants in the NTIS group, the renal dysfunction group, and the normal group (Fig. 2c).

### Comparison of mortality rate among the four groups

**Fig. 3:**
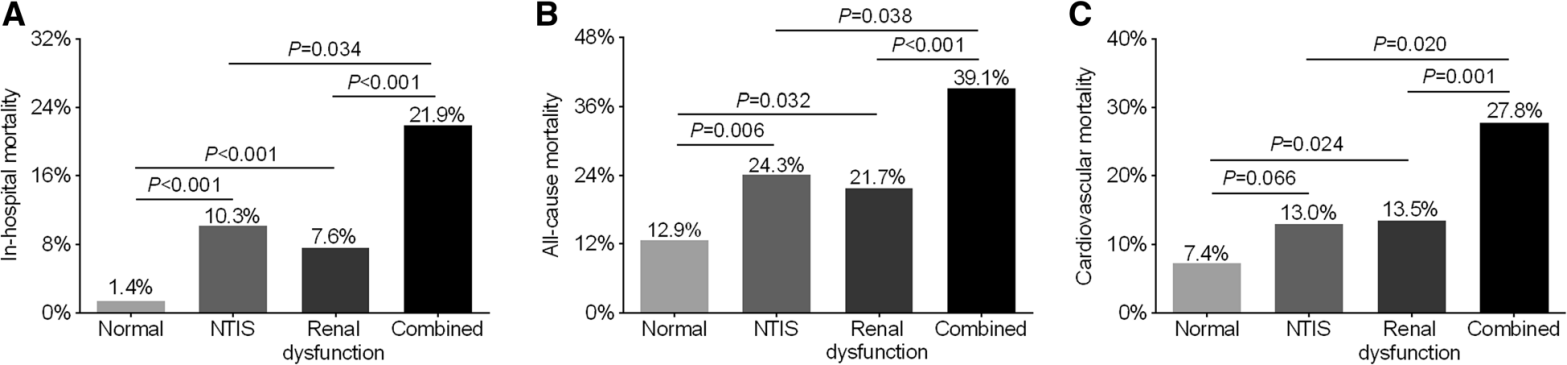
Comparison of in-hospital, all-cause and cardiovascular mortality among the four groups. (**a**) In-hospital mortality among the four groups. (**b**) All-cause mortality among the four groups. (**c**) Cardiovascular mortality among the four groups. Overall and cardiovascular mortality did not include in-hospital mortality

The comparison of mortality rate in the four groups is demonstrated in Fig. 3. In-hospital mortality was 21.9% in the patients with combined NTIS and renal dysfunction, which was significantly higher than that in the NTIS group (10.3%, *P* = 0.034), the renal dysfunction group (7.6%, *P* < 0.001), and the normal group (1.4%, *P* < 0.001) (Fig. 3a). During a median follow-up of 4 years, 213 patients died, which included 130 patients due to cardiovascular causes. Similar to in-hospital mortality, all-cause mortality and cardiovascular mortality in the combined group were 39.1 and 27.8%, respectively, which were also significantly higher than in the NTIS group (24.3%, *P* = 0.038; 13.0%, *P* = 0.020, respectively), the renal dysfunction group (21.7%, *P* < 0.001; 13.5%, *P* = 0.001, respectively) and the normal group (12.9%, *P* < 0.001; 7.4%, *P* < 0.001, respectively) (Fig. 3b and Fig. 3c).

### Comparison of mortality risk among the four groups

A binary logistic regression analysis associated with in-hospital mortality is shown in Table 2. When the normal group was considered as reference, the combined group, the NTIS group, and the renal dysfunction group showed an obviously increased risk for in-hospital death(OR: 7.798, 95% CI: 2.722 to 22.339, *P* < 0.001; OR: 3.643, 95% CI: 1.154 to 11.505, *P* = 0.028; and OR: 3.135, 95% CI: 1.043 to 9.422, *P* = 0.042, respectively). Compared to those in the NTIS and the renal dysfunction group, the patients with combined NTIS and renal dysfunction also showed an increased risk for in-hospital mortality (OR: 2.916, 95% CI: 1.054 to 8.066, *P* = 0.039 versus NTIS group; OR: 2.487, 95% CI: 1.064 to 5.816, *P* = 0.036 versus renal dysfunction group, respectively).

**Table 2:** Comparison of Odds Ratio of in-hospital mortality

	Normal Group	NTIS Group			Renal dysfunction Group			Combined Group		
	OR (95% CI)	OR	95% CI	*P* value	OR	95% CI	*P* value	OR	95% CI	*P* value
Model 1	1	7.034	3.019–16.386	< 0.001	4.155	1.889–9.141	< 0.001	14.065	6.469–30.578	< 0.001
Model 2	1	4.256	1.383–13.096	0.012	5.445	2.023–14.657	0.001	7.778	2.688–22.508	< 0.001
Model 3	1	3.643	1.154–11.505	0.028	3.135	1.043–9.422	0.042	7.798	2.722–22.339	< 0.001

Model1: Adjusted for age, sex, smoking use, alcohol status, hypertension, diabetes, medical therapy (use of antiplatelet agents, β-Blockers, LLDs, ACEIs/ARBs, CCBs, and Diuretics), and BMI (for all patients)

Model2: Adjusted for age, sex, smoking use, alcohol status, hypertension, diabetes, medical therapy, BMI, LVEF, Killip class, lg(NT-pro BNP), infarct type (NSTEMI vs STEMI), prior PCI or CABG and revascularization (PCI, CABG) (for all patients)

Model3: Adjusted for age, sex, smoking use, alcohol status, hypertension, diabetes, medical therapy, BMI, LVEF, Killip class, lg(NT-pro BNP), infarct type (NSTEMI vs STEMI), prior PCI or CABG and revascularization (PCI, CABG), WBC, Hb, Alb, TC, TG, HDL-c, LDL-c, FPG and CRP (for all patients)

**Fig. 4:**
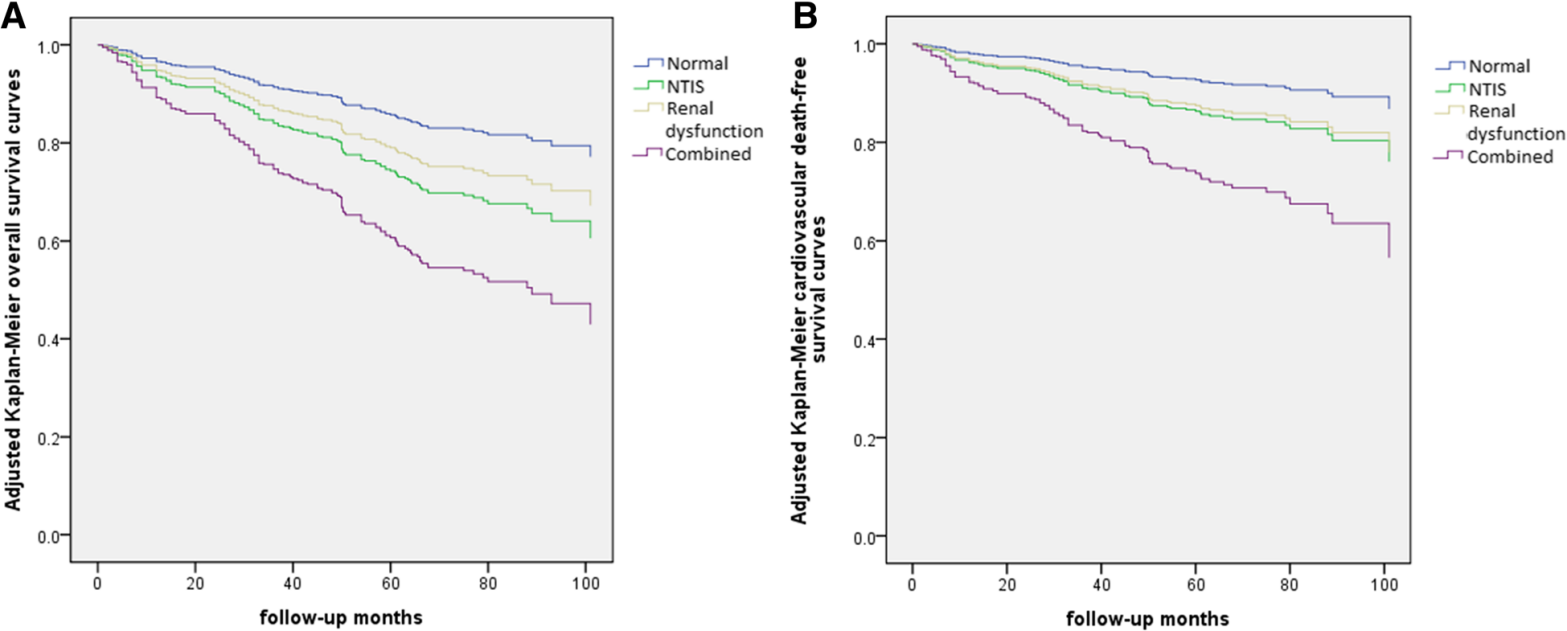
Adjusted Kaplan-Meier survival curves among the four groups. (**a**) Adjusted Kaplan-Meier overall survival curves. (b) Adjusted Kaplan-Meier cardiovascular death-free survival curves. Adjusted for age, sex, smoking use, alcohol status, hypertension, diabetes mellitus, medical therapy (use of antiplatelet agents, β-Blockers, LLDs, ACEIs/ARBs, CCBs, and Diuretics), and BMI

A Cox proportional hazard regression analysis associated with long-term all-cause and cardiovascular mortality is shown in Table 3. Compared to the normal group, the combined group had 4.140 times the risk of all-cause mortality (95% CI: 2.534 to 6.764; *P* < 0.001); the NTIS group had 2.138 times (95% CI: 1.234 to 3.704; *P* = 0.007) and the renal dysfunction group had 2.050 times the risk of overall mortality (95% CI: 1.271 to 3.307; *P* = 0.003). The combined group, the NITS group and the renal dysfunction group had also an increased risk of cardiovascular mortality compared with the normal group (HR: 5.152, 95% CI: 2.786 to 9.527, *P* < 0.001; HR: 2.134, 95% CI: 1.028 to 4.430, *P* = 0.042; HR: 2.237, 95% CI: 1.210 to 4.135, *P* = 0.010, respectively). In addition, compared with the NTIS group and the renal dysfunction group, the combined group represented a higher risk of all-cause mortality (HR: 1.939, 95% CI: 1.139 to 3.304, *P* = 0.015 versus NTIS group; HR: 2.020, 95% CI: 1.300 to 3.140, *P* = 0.002 versus renal dysfunction group, respectively) and cardiovascular mortality (HR: 2.420, 95% CI: 1.239 to 4.725, *P* = 0.010 versus NTIS group; HR: 2.303, 95% CI: 1.373 to 3.862, *P* = 0.002 versus renal dysfunction group, respectively). The adjusted Kaplan-Meier curves demonstrated that overall survival and cardiovascular death-free survival in the patients with combined NTIS and renal dysfunction were obviously lower than those in the NTIS group, the renal dysfunction group, and the normal group (all *P* < 0.05) (Fig. 4).

**Table 3:** Comparison of Hazard Ratio of all-cause and cardiovascular mortality

	Normal Group	NTIS Group			Renal dysfunction Group			Combined Group		
	HR (95% CI)	HR	95% CI	*P* value	HR	95% CI	*P* value	HR	95% CI	*P* value
All-cause mortality										
Model 1	1	1.912	1.219–2.998	0.005	1.514	1.056–2.170	0.024	3.209	2.157–4.774	< 0.001
Model 2	1	2.036	1.252–3.312	0.004	1.675	1.090–2.572	0.019	3.488	2.223–5.471	< 0.001
Model 3	1	2.138	1.234–3.704	0.007	2.050	1.271–3.307	0.003	4.140	2.534–6.764	< 0.001
Cardiovascular mortality										
Model 1	1	1.903	1.010–3.587	0.047	1.730	1.074–2.788	0.024	3.955	2.387–6.553	< 0.001
Model 2	1	2.141	1.087–4.217	0.028	2.089	1.185–3.684	0.011	4.580	2.578–8.136	< 0.001
Model 3	1	2.134	1.028–4.430	0.042	2.237	1.210–4.135	0.010	5.152	2.786–9.527	< 0.001

Model1: Adjusted for age, sex, smoking use, alcohol status, hypertension, diabetes, medical therapy (use of antiplatelet agents, β-Blockers, LLDs, ACEIs/ARBs, CCBs, and Diuretics), and BMI (for all patients)

Model2: Adjusted for age, sex, smoking use, alcohol status, hypertension, diabetes, medical therapy, BMI, LVEF, Killip class, lg(NT-pro BNP), infarct type (NSTEMI vs STEMI), prior PCI or CABG and revascularization (PCI, CABG) (for all patients)

Model3: Adjusted for age, sex, smoking use, alcohol status, hypertension, diabetes, medical therapy, BMI, LVEF, Killip class, lg(NT-pro BNP), infarct type (NSTEMI vs STEMI), prior PCI or CABG and revascularization (PCI, CABG), WBC, Hb, Alb, TC, TG, HDL-c, LDL-c, FPG and CRP (for all patients)

The sensitivity analysis showed that the combined renal dysfunction (eGFR was calculated with CKD-EPI formula) and NTIS also further increased in-hospital mortality(OR: 7.139, 95% CI: 2.554 to 19.954, *P* < 0.001 versus normal group; OR: 2.637, 95% CI: 0.993 to 7.003, *P* = 0.052 versus NTIS group; OR: 3.178, 95% CI: 1.327 to 7.609, *P* = 0.009 versus renal dysfunction group, respectively), all-cause mortality(HR: 3.616, 95% CI: 2.172 to 6.021, *P* < 0.001 versus normal group; HR: 1.817, 95% CI: 1.071 to 3.084, *P* = 0.027 versus NTIS group; HR: 1.916, 95% CI: 1.180 to 3.109, *P* = 0.009 versus renal dysfunction group, respectively) and cardiovascular mortality(HR: 4.790, 95% CI: 2.539 to 9.036, *P* < 0.001 versus normal group; HR: 2.425, 95% CI: 1.071 to 3.084, *P* = 0. 010 versus NTIS group; HR: 2.118, 95% CI: 1.244 to 3.608, *P* = 0. 006 versus renal dysfunction group, respectively) (Additional file 1: Table 1 and Additional file 2: Table 2).

## Discussion

Despite some controversy, both NTIS and renal dysfunction levels were widely considered to be associated with increased mortality risk in AMI patients. Most studies have supported the long-term prognostic role of NTIS and renal dysfunction in AMI [8, 12, 13, 15]. It is well-known that NTIS is associated with increased inflammatory burden and vascular resistance, and decreases heart rate as well as cardiac contractility, which reduce cardiac output and further accelerate the progression of ischemic heart disease [1, 18, 30, 31]. Altay et al. [32] also indicated that inflammatory status of the upper normal TSH tertile correlated negatively with FT3 in women may mediate mortality or nonfatal cardiac events rather than the action of thyroid hormone. Therefore, NTIS has been suggested to be associated with increased overall and cardiovascular death [5–9]. Similarly, glomerular hyperfiltration is associated with autoimmune activation and low-grade inflammation, leading to chronic diseases like ischemic heart disease [33]. Renal dysfunction has also been related to dyslipidemia, atherosclerosis, arterial calcification and cardiac dysfunction in the progression of inflammation [14, 16, 30]. It was suggested that patients with severe renal insufficiency had 8 times the risk of cardiovascular mortality compared with general population [34]. Consistent with these studies, our findings also showed that both NTIS and renal dysfunction were closely associated with increased mortality risk in AMI patients. Compared with the patients in the normal group, the patients with NTIS had nearly 3.6-, 2.1- and 2.1- fold risk of in-hospital, all-cause and cardiovascular mortality, respectively. Similarly, the patients with renal dysfunction had also obviously increased mortality risk.

However, it has not been investigated so far whether the coexistence of NTIS and renal dysfunction further increases mortality risk in patients with AMI. Therefore, we investigated the prognostic role of combined NTIS and renal dysfunction in AMI. The present findings verified our hypothesis that the patients with combined NTIS and renal dysfunction had a significantly reduced cardiac function and poorer prognosis with significantly increased mortality risk compared with the other three groups. Although only 12.4% of the AMI patients had combined NTIS and renal dysfunction, it accounted for almost half of all deaths within follow-up periods.

Therefore, it is evident that there exists a close association between thyroid and renal function. Thyroid hormone alterations are often seen with the worsening of kidney function. Pathophysiological factors caused by renal insufficiency contribute to the development of NTIS. The kidney is associated with the metabolism and excretion of thyroid hormone [35]. Renal insufficiency-related metabolic disorders such as consumption of various proteins, metabolic acidosis, anemia and iodide retention inhibit the forming, releasing and converting of thyroid hormone, which is strongly associated with the presence of NTIS [18]. It was found that NTIS state was present in more than 75% of individuals with end-stage renal disease [18, 20]. Furthermore, Song et al. [20] reported that T3 levels were positively associated with eGFR levels. On the other hand, thyroid hormone also plays an important role in preserving normal renal function. Thus, NTIS can also result in deterioration of renal function via several pathways including renal hypoperfusion resulted from worsening cardiac output, activation of the renin angiotensin and aldosterone pathways (RAAS), reduction of systemic levels of vasodilators and decrease of reabsorption of sodium and water [18]. The fact in this study that NTIS was more frequently detected in patients with renal dysfunction than preserved renal function, and renal dysfunction was more common in patients with thyroid dysfunction than normal thyroid profile supported the close association between thyroid hormone and renal function.

Furthermore, it is suggested that NTIS and renal dysfunction contribute to the deterioration of cardiac function. Studies reported that NTIS caused deterioration of cardiac function early after AMI and further ventricular remodeling, as well as arrhythmia, vasoconstriction, dyslipidemia, and vascular calcification [1, 18, 19]. It was reported that NTIS was associated with lower LVEF and cardiac index [5, 19]. Likewise, renal functional impairment contributed to the progression of left ventricular hypertrophy, increase in cardiac filling pressures and progressive ventricular dilation by sodium and water retention [16, 36]. It was found 85% of patients with end-stage renal disease having abnormal left ventricular structure and function [36]. Consistent with these findings, we also found that compared with those with normal thyroid and renal function, the patients with either NTIS or renal dysfunction had significantly poorer cardiac function, such as lower LVEF and higher NT-pro BNP.

More importantly, the combination of NTIS and renal dysfunction further aggravates heart function, and increases mortality risk in AMI patients in the present study. The fact that NTIS is associated with lower LVEF in patients with renal insufficiency may also implicate the combined adverse effect of NTIS and renal dysfunction on heart function [37]. The interactions between NTIS and renal dysfunction cause deterioration of cardiac function. We found that the patients with combined NTIS and renal dysfunction had markedly lower LVEF, higher percentage of Killip class>IIand higher NT-pro BNP levels than the patients with NTIS and the patients with renal dysfunction. It implied that combined NTIS and renal dysfunction was associated with more serious deterioration of cardiac function than either NTIS or renal dysfunction in AMI patients. More importantly, the combination of NTIS and renal dysfunction strongly predicted short term in-hospital mortality, long term all-cause and cardiovascular mortality in AMI, which were far in excess of that observed AMI patients with either NTIS or renal dysfunction alone. Therefore, our findings may verify that the combination of NTIS and renal dysfunction is a strong independent predictor of mortality in patients with AMI, exceeding either isolated NTIS or isolated renal dysfunction, possibly through the deterioration of cardiac function. Our findings suggest that in AMI patients, renal dysfunction and nonthyroidal illness syndrome represent a combination of disease progression. NTIS and kidney dysfunction may mutually amplify their respective impacts on overall and cardiovascular mortality in AMI, which cause a synergistic action and finally remarkably increase mortality risks in patients with AMI. Meanwhile, cardiac ischemia and dysfunction may cause progression of renal failure and presence of NTIS [1, 16, 18, 38]. Worsening renal function was common in heart failure patients in the post-MI period [38, 39]. Therefore, NTIS, renal dysfunction, and reduced heart function may form a vicious circle in AMI, which lead to a further increase of mortality.

Some limitations should be acknowledged. First, this is a single-center cohort study, in which basal characteristics of groups is not equal. However, we have controlled these confounding factors as much as possible in the present analyses. Despite the inherent design limitations, patients in observational studies better represent those seen in clinical practice. In addition, the present study lacks data of follow-up thyroid and renal function tests. Moreover, with 6.4% of total patients lost to follow up, data on the endpoint outcome is not totally complete. The date range of enrollment is extended in order to increase the sample size and Cox proportional hazard regression analyses have been applied to reduce bias. A far larger multi-center prospective cohort study should be carried out to verify our findings in AMI population and other populations.

## Conclusions

The present findings suggest that both NTIS and renal dysfunction increase short-term in-hospital mortality, and long-term all-cause and cardiovascular mortality risk in patients with AMI. Furthermore, the coexistence of NTIS and renal dysfunction presents further reduced heart function, and predicts further increased mortality risk in patients with AMI.

## Additional files

Additional file 1: **Table S1.** Comparison of Odds Ratio of in-hospital mortality when eGFR was calculated with CKD-EPI formula. (DOC 32 kb)

Additional file 2: **Table S2.** Comparison of Hazard Ratio of all-cause and cardiovascular mortality when eGFR was calculated with CKD-EPI formula. (DOCX 20 kb)

## Abbreviations

ACEIs: Angiotensin-Converting Enzyme Inhibitors
Alb: Albumin
AMI: Acute Myocardial Infarction
ARBs: Angiotensin Receptor Blockers
BMI: Body Mass Index
CABG: Coronary Artery Bypass Graft
CCBs: Calcium Channel Blockers
CRP: C-reactive Protein
DBP: Diastolic Blood Pressure
eGFR: Estimated Glomerular Filtration Rate
FPG: Fasting Plasma Glucose
FT3: Free Triiodothyronine
FT4: Free Thyroxine
Hb: Hemoglobin
HDL-C: High Density Lipoproteins Cholesterol
LDL-C: Lower Density Lipoproteins Cholesterol
LLDs: Lipid-Lowering Drugs
LVEF: Left Ventricular Ejection Fraction
NSTEMI: Non ST-segment Elevation Myocardial Infarction
NTIS: Nonthyroidal Illness Syndrome
NT-Pro BNP: N-terminal Pro-brain Natriuretic Peptide
PCI: Percutaneous Coronary Intervention
SBP: Systolic Blood Pressure
sCr: Serum Creatinine
STEMI: ST-segment Elevation Myocardial Infarction
TC: Total Cholesterol
TG: Total Triglyceride
TSH: Thyroid-Stimulating Hormone
WBC: White Blood Cell
