# Supplementary material for: The combination of nonthyroidal illness syndrome and renal dysfunction further increases mortality risk in patients with acute myocardial infarction: a prospective cohort study
Source: BMC Cardiovasc Disord. 2019 Mar 4;19:50. doi: 10.1186/s12872-019-1027-1 (PMC6398216; doi:10.1186/s12872-019-1027-1)
Supplement: Supplementary file 1 — Table S1. Comparison of Odds Ratio of in-hospital mortality when eGFR was calculated with CKD-EPI formula. (DOC 32 kb) [file 12872_2019_1027_MOESM1_ESM.doc]

**Supplementary Table** **1 Comparison of Odds Ratio of in-hospital mortality when eGFR was calculated with CKD-EPI formula**

|  | **Normal Group(n=707)** |  |  | **NTIS Group(n=139)** | |  | **renal dysfunction Group(n=289)** | | |  | **Combined Group(n=160)** | | |
| --- | --- | --- | --- | --- | --- | --- | --- | --- | --- | --- | --- | --- | --- |
|  | OR (95% CI) |  | OR | 95% CI | p value |  | OR | 95% CI | p value |  | OR | 95% CI | p value |
| Model 1 | 1 |  | 6.103 | 2.673-13.935 | <0.001 |  | 3.806 | 1.746-8.298 | 0.001 |  | 14.036 | 6.556-30.048 | <0.001 |
| Model 2 | 1 |  | 3.175 | 1.060-9.506 | 0.039 |  | 4.470 | 1.712-11.670 | 0.002 |  | 7.667 | 2.737-21.473 | <0.001 |
| Model 3 | 1 |  | 2.708 | 0.883-8.312 | 0.082 |  | 2.246 | 0.744-6.783 | 0.151 |  | 7.139 | 2.554-19.954 | <0.001 |

Model1: Adjusted for age, sex, smoking use, alcohol status, hypertension, diabetes, medical therapy (use of antiplatelet agents, β-Blockers, LLDs, ACEIs/ARBs, CCBs, and Diuretics), and BMI (for all patients)

Model2: Adjusted for age, sex, smoking use, alcohol status, hypertension, diabetes, medical therapy, BMI, LVEF, Killip class, lg(NT-pro BNP), infarct type (NSTEMI vs STEMI) , prior PCI or CABG and revascularization (PCI, CABG) (for all patients)

Model3: Adjusted for age, sex, smoking use, alcohol status, hypertension, diabetes, medical therapy, BMI, LVEF, Killip class, lg(NT-pro BNP), infarct type (NSTEMI vs STEMI) , prior PCI or CABG and revascularization (PCI, CABG), WBC, Hb, Alb, TC, TG, HDL-c, LDL-c, FPG and CRP (for all patients)
