# Supplementary material for: The combination of nonthyroidal illness syndrome and renal dysfunction further increases mortality risk in patients with acute myocardial infarction: a prospective cohort study
Source: BMC Cardiovasc Disord. 2019 Mar 4;19:50. doi: 10.1186/s12872-019-1027-1 (PMC6398216; doi:10.1186/s12872-019-1027-1)
Supplement: Supplementary file 2 — Table S2. Comparison of Hazard Ratio of all-cause and cardiovascular mortality when eGFR was calculated with CKD-EPI formula. (DOCX 20 kb) [file 12872_2019_1027_MOESM2_ESM.docx]

**Supplementary Tab****le 2 Comparison of Hazard Ratio of all-cause and cardiovascular mortality when eGFR was calculated with CKD-EPI formula**

|  | **Normal Group(n=652)** | |  |  | **NTIS Group(n=115)** | |  | **renal dysfunction Group(n=252)** | | |  | **Combined Group(n=115)** | | |
| --- | --- | --- | --- | --- | --- | --- | --- | --- | --- | --- | --- | --- | --- | --- |
|  | | HR (95% CI) |  | HR | 95% CI | *P* value |  | HR | 95% CI | *P* value |  | HR | 95% CI | *P* value |
| **All-cause mortality** | |  |  |  |  |  |  |  |  |  |  |  |  |  |
| Model 1 | | 1 |  | 2.226 | 1.467-3.377 | <0.001 |  | 1.450 | 1.006-2.091 | 0.046 |  | 2.788 | 1.842-4.221 | <0.001 |
| Model 2 | | 1 |  | 2.352 | 1.493-3.705 | <0.001 |  | 1.603 | 1.038-2.475 | 0.033 |  | 3.030 | 1.897-4.840 | <0.001 |
| Model 3 | | 1 |  | 2.556 | 1.530-4.271 | <0.001 |  | 1.916 | 1.180-3.109 | 0.009 |  | 3.616 | 2.172-6.021 | <0.001 |
| **Cardiovascular mortality** | | |  |  |  |  |  |  |  |  |  |  |  |  |
| Model 1 | | 1 |  | 2.341 | 1.318-4.158 | 0.004 |  | 1.644 | 1.014-2.666 | 0.044 |  | 3.443 | 2.041-5.809 | <0.001 |
| Model 2 | | 1 |  | 2.688 | 1.437-5.028 | 0.002 |  | 2.085 | 1.178-3.689 | 0.012 |  | 4.086 | 2.258-7.394 | <0.001 |
| Model 3 | | 1 |  | 2.679 | 1.356-5.292 | 0. 005 |  | 2.261 | 1.215-4.207 | 0.010 |  | 4.790 | 2.539-9.036 | <0.001 |

Model1: Adjusted for age, sex, smoking use, alcohol status, hypertension, diabetes, medical therapy (use of antiplatelet agents, β-Blockers, LLDs, ACEIs/ARBs, CCBs, and Diuretics), and BMI (for all patients)

Model2: Adjusted for age, sex, smoking use, alcohol status, hypertension, diabetes, medical therapy, BMI, LVEF, Killip class, lg(NT-pro BNP), infarct type (NSTEMI vs STEMI) , prior PCI or CABG and revascularization (PCI, CABG) (for all patients)

Model3: Adjusted for age, sex, smoking use, alcohol status, hypertension, diabetes, medical therapy, BMI, LVEF, Killip class, lg(NT-pro BNP), infarct type (NSTEMI vs STEMI) , prior PCI or CABG and revascularization (PCI, CABG), WBC, Hb, Alb, TC, TG, HDL-c, LDL-c, FPG and CRP (for all patients)
